# Treated post-acute sequelae after COVID-19 in a German matched cohort study using routine data from 230,256 adults

**DOI:** 10.3389/fepid.2022.1089076

**Published:** 2023-02-14

**Authors:** Doreen Müller, Sandra Stengel, Martin Roesler, Gerhard Schillinger, Hendrik Dräther, Christian Günster, Hanna Tillmanns, Michael Erhart, Joachim Szecsenyi, Uta Merle

**Affiliations:** ^1^ AOK Research Institute, Berlin, Germany; ^2^ Department of General Practice and Health Services Research, Heidelberg University Hospital, Marsilius Arkaden, Turm West, Heidelberg, Germany; ^3^ AOK-Bundesverband, Berlin, Germany; ^4^ Department for Health and Rehabilitation Sciences, Alice-Salomon-Hochschule Berlin, Berlin, Germany; ^5^ Psychology Department, Apollon Hochschule der Gesundheitswirtschaft, Bremen, Germany; ^6^ Clinic for Gastroenterology, Infectious Diseases, Toxicology, Heidelberg University Hospital, Heidelberg, Germany

**Keywords:** SARS-CoV-2, COVID-19, post-acute sequelae, long covid, post covid

## Abstract

**Background:**

Post-acute sequelae after COVID-19 are still associated with knowledge gaps and uncertainties at the end of 2022, e.g., prevalence, pathogenesis, treatment, and long-term outcomes, and pose challenges for health providers in medical management. The aim of this study was to contribute to the understanding of the multi-faceted condition of long-/ post-COVID. It was designed to evaluate whether a prior SARS-CoV-2 infection during the first COVID-19 wave in Germany increases the rate of disease, as measured *via* a record of insurance data on diagnoses, symptoms, and treatment, in the subsequent 12 months compared with matched control groups without recorded SARS-CoV-2 infection.

**Method:**

50 outcome variables at disease, symptom and treatment levels (14 main categories and 36 sub-categories; new diagnoses) were defined from health insurance data. Logistic regression was carried out for two groups of patients tested positive in a PCR test in March/April 2020 for SARS-CoV-2, compared to the respective risk-adjusted (age, administrative region, 1:5 propensity-score matching), contemporaneous control group without prior documented SARS-CoV-2 infection (CG): First, individuals with outpatient treatment of acute COVID-19, indicating a not severe course (COV-OUT), and second, individuals with inpatient treatment of acute COVID-19, indicating a severe course (COV-IN) were compared with their respective control group.

**Results:**

The mortality rate in COV-OUT (*n* = 32,378) and COV-IN (*n* = 5,998) groups is higher compared to their control groups with odds ratio (OR) 1.5 [95%CI (1.3, 1.6)] and 1.7 [95%CI (1.5, 1.8)] respectively. Both groups were more likely to have experienced at least one outcome compared to their CG [OR = 1.4, 95%CI (1.4, 1.4)]; OR = 2.5, 95%CI [2.4, 2.6]). 42/37 (COV-IN/COV-OUT) outcome variables showed increased ORs. COV-OUT: Loss of taste and smell [OR = 5.8, 95%CI (5.1, 6.6)], interstitial respiratory diseases [OR = 2.8, 95%CI (2.0, 4.1)] and breathing disorders [OR = 3.2, 95%CI (2.2, 4.7)] showed the highest ORs. COV-IN: Interstitial respiratory diseases [OR = 12.2, 95%CI (8.5, 17.5)], oxygen therapy [OR = 8.1, 95%CI (6.4, 10.2)] and pulmonary embolism/anticoagulation [OR = 5.9, 95%CI (4.4, 7.9)] were the most pronounced.

**Conclusion:**

Following a SARS-CoV-2 infection during the first wave of the COVID-19 pandemic in Germany, 8.4 [COV-OUT, 95%CI (7.7, 9.1)] respectively 25.5 [COV-IN, 95%CI (23.6, 27.4)] percentage points more subjects showed at least one new diagnosis/symptom/treatment compared to their matched CG (COV-OUT: 44.9%, CG: 36.5%; COV-IN: 72.0%, CG: 46.5%). Because the symptoms and diagnoses are so varied, interdisciplinary and interprofessional cooperation among those providing management is necessary.

## 1. Introduction

In retrospect, the COVID-19 pandemic is described in different phases, whereby the first COVID-19 wave lasted from calendar week 10 to 20/2020, further COVID-19 waves followed (1). Modifications in the pathogen characteristics led to the emergence of virus variants (2). In Germany, more than 36 million SARS-CoV-2-positive cases were registered with the Federal Institute for Public Health (Robert Koch Institute), with 157,495 mortalities, corresponding to a case mortality rate of 0.43% (as per November 28 2022) (3). Sequelae after acute COVID-19 were initially referred to as long COVID in 2020 (4). In the meantime, research is being carried out worldwide into post-acute sequelae after COVID-19, whereby knowledge gaps and uncertainties concerning, for example, prevalence, pathogenesis, treatment as well as the long-term effects present healthcare professionals with coordinative, organisational and financial challenges concerning the medical management (5–8).

Currently, different symptom-based definitions of long- /post-COVID exist. The National Institute for Health and Care Excellence categorises health problems that occur up to four weeks after the beginning of the disease as acute COVID, and between 4 and 12 weeks as “persistent COVID-19”/ long-COVID and from the 12th week congruent with the clinical case definition by the World Health Organisation (WHO) as post-COVID syndrome (9–11).

Data on the prevalence of sequelae following SARS-CoV-2 are heterogeneous and mostly based on non-controlled studies in which mainly symptoms were enquired about, or coded diagnoses were described without recording treatment needs such as administering medicines or prescribing therapies (12, 13).

According to the lack of objective parameters to diagnose and identify post-COVID as of today, diagnoses are based on symptoms. The WHO identified fatigue, shortness of breath, neurocognitive and other symptoms as common post-COVID symptoms (11). Fatigue, dyspnoea, sleep disorders and myalgias are described as the most common symptoms persisting 12 months after infection (12), whereas fatigue, neurocognitive impairment, chest symptoms are prevalent after 6 to 12 months (14). Neuropsychiatric symptoms, pulmonary, liver, heart and kidney disorders, thrombosis, stroke, and embolism (15) were identified in a meta-analysis as additional post-acute COVID-19 sequelae. Analyses of follow-up routine data from hospitalised patients showed an increased risk of morbidity, mortality, and hospital readmission (16, 17).

The aim of the study was to evaluate whether a prior SARS-CoV-2 infection during the first COVID-19 wave in Germany increases the rate of disease, as measured *via* a record of insurance data on diagnoses, symptoms, and treatment, in the subsequent 12 months compared with matched control groups without recorded SARS-CoV-2 infection. Furthermore, sex differences were also to be investigated. The analysis is based on health insurance data of the AOK, Germany's largest statutory health insurance, which contains information on the use of health care facilities, including diagnoses and treatments from billing data.

## 2. Method

### 2.1. Study design and setting

**Figure 1:**
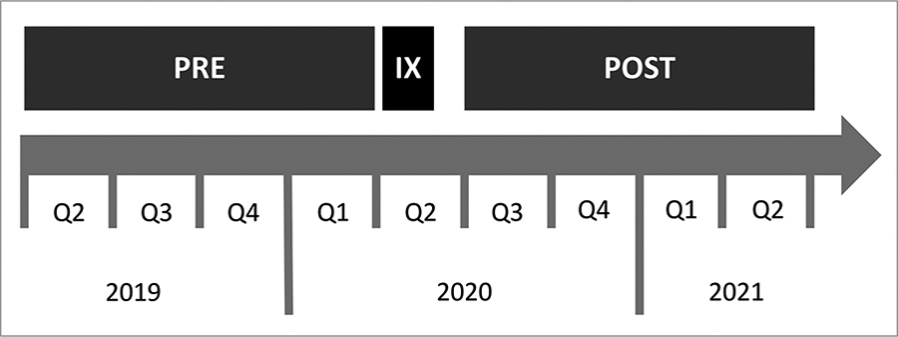
Study design. Notes: PRE: Pre-observation period (April 2019 – March 2020); IX: Index period (April – May 2020) *; POST: Post-observation period (July 2020 – June 2021). * Context: The Robert Koch Institute issued the recommendation for every acute respiratory symptom (irrespective of their severity) to be tested for COVID-19 as of March 24 2020. The sentence “without known risk factors (COVID-19 diagnostic only if enough testing capacity available)” was deleted as of April 22 2020 (25). In the index period the prevalent virus variant in Germany was the wild type (2).

A matched cohort study was carried out of persons insured with the AOK, who were treated for COVID-19 as outpatients or inpatients. The AOK is the largest German statutory health insurance, covering about 30 per cent of the German population. Germany-wide billing data from outpatient and hospital care, prescriptions of medicines, medical aids and remedies, as well as the master data of AOK-insured persons for the period from April 2019 to June 2021 (index period) were analysed. Data of patients who had a positive polymerase chain reaction (PCR) test for SARS-CoV-2 were observed for twelve months (equivalent to four quarters) after infection (post-observation period), beginning 4 weeks after the COVID-19 diagnosis, which corresponds to the usual definition of post-acute sequelae after COVID-19 (9, 10). In order to detect new (incident) cases or worsening diseases in the post-observation period, patients data were pre-observed across twelve months before their COVID-19 diagnosis (pre-observation period) (see Figure 1).

#### 2.1.1. Sampling

Insured persons aged 18–99 years were selected if they had been insured with the AOK without interruption during the pre-observation period until the start of post-observation and were then continuously insured either for the entire post-observation period or until their death (if this occurred before that). Four groups were distinguished: (1) The “COVID-19 outpatient” group included those who had a first outpatient diagnosis of U07.1 according to ICD-10-GM in April/May 2020 (first wave of the COVID-19 pandemic in Germany), had made use of an outpatient SARS-CoV-2 PCR test, and had not been treated in hospital between April-June 2020, indicating a not severe course of acute COVID-19 (COV-OUT). (2) The “COVID-19 inpatient” group included those who were treated for COVID-19 for the first time as inpatients in April/May 2020, indicating a severe course of acute COVID-19 (COV-IN). Cases with a principal diagnosis of respiratory failure, pulmonary embolism, viral infection, sepsis or renal failure and a secondary diagnosis of U07.1 were included in line with Guenster et al. (17), since the COVID-19 diagnosis cannot be documented as a principal diagnosis in inpatient billing data. 3 and 4) For each of these groups, a contemporaneous control group was formed of persons who visited their general practitioner at least once in April/May 2020 (non-users excluded), had no hospitalisation in April-June 2020 and had no COVID-19-related diagnosis during the entire observation period. Matching was used to form two risk-adjusted control groups for the two COVID-19 cohorts.

### 2.2. Outcome variables

Based on different factors described in the literature (15–24), 14 outcome categories consisting of 50 outcomes were defined in an interdisciplinary and interprofessional consensus process within the research group. The outcomes included new cases of acute disease, chronic diseases, symptoms, prescriptions of medicines, medical aids and remedies, as well as psychotherapy treatments and death.

Treated long COVID symptoms were operationalised by means of data referring to specific services covered by statutory health insurance, for example, respiratory disorders by means of medication or respiratory therapy, cardiac symptoms by cardiac co-treatments, thyroid diseases by medication (Supplement 1). The outcomes were operationalised as binary data (yes/no) and indicate whether an event occurred for the first time or worsened (renal insufficiency, hypertension) during the post-observation period compared to the pre-observation period (Supplement 1). The assignment of a nursing-care-dependency level in the pre-observation period was recorded as a binary variable (yes/no) in order to perform an ex-post analysis.

### 2.3. Matching

To form the control groups, a two-stage matching procedure was defined and employed resulting in five control group matches for each individual in the two COVID-19 groups. For 38.376 individuals included in the COVID-19 groups, roughly 8 million individuals where available to choose statistical matches from.

First, an exact matching was carried out in the program R (version 4.1.1) with regard to age in years and the four-stage settlement structure of the administrative region of the insured person's place of residence. Age is described as a risk factor for developing post-acute symptoms after COVID-19 (25). The administrative region used in spatial research (26) provides information about the different accessibilities of outpatient and inpatient healthcare facilities between regions.

Secondly, propensity score matching was applied for morbidity-related risk factors for severe COVID-19 courses that existed in the pre-observation period. The 35 disease groups specified in Roessler et al. (27) were used. The propensity score was determined using logistic regression and an optimal matching procedure was implemented using the R package optmatch (version 0.10.5).

The evaluation of standardized differences showed a very high degree of balance in the matched samples, due to the vast pool of possible matches. Therefore, the propensity score was not used for double adjustment in the subsequent logistic regressions.

### 2.4. Statistical analyses

For the description of the basic characteristics of the study groups as well as the distribution of the outcome variables, proportion values are given for categorical variables and mean and standard deviation for continuous variables. The effect of COVID-19 on each of the 50 outcome variables was estimated using logistic regressions for both the outpatient COVID group and the associated control group, as well as the inpatient COVID and control group. In order to identify sex differences, the regression models were extended to include the explanatory variable sex and the interaction sex*COVID-19. The propensity score was determined using logistic regression and an optimal matching procedure was implemented using the R package optmatch (version 0.10.5). If the interaction is significant, the effect of COVID-19 differs between women and men. In these cases, the COVID effect was ultimately estimated individually for women and men using logistic regression. Only outcome variables with a frequency (also within the sex categories) greater than or equal to 10 persons (28) were considered. In a sensitivity analysis, the additional influence of a pre-existing nursing-care dependency was examined. The data transformations and analyses were carried out using SQL and R (version 4.1.1). The reporting is based on the RECORD checklist (29).

## 3. Results

### 3.1. Description of the study population

Table 1 shows the characteristics of the study population. A total of 8,392,550 insured persons were included, of which 32,378 were in the COVID-19 outpatient group and 5,998 in the COVID-19 inpatient group. The outpatient COVID-19 group, aged an average of 48.4 years, is significantly younger than the inpatient COVID-19 group, with an average of 66.6 years. The proportion of women is higher in the outpatient COVID-19 group at 61.6% than in the inpatient COVID-19 group at 48.1%. In all groups, the urban administrative region is the most common. For all insured persons with COVID-19, control persons could be determined in a ratio of 1:5. Based on the 35 risk factors for a severe COVID-19 course according to Roessler (27), as well as taking into consideration the assignment of a nursing-care dependency, it becomes apparent that the two COVID-19 groups are more similar to their respective matched control group than to the unmatched control group.

**Table 1:** Characteristics of the study population.

	Potential control group sample^a^	COVID-19 outpatient group	Matched control group outpatient	COVID-19 inpatient group	Matched control group inpatient
	(w/o matching)	(after matching)		(after matching)	
Number of subjects: *n*					
Total	8,392,550	32,378	161,890	5,998	29,990
Subjects non-deceased	8,204,079	31,610	159,224	5,535	28,542
Demographics					
Age: Mean (standard deviation)	60.1 (18.6)	48.4 (18.7)	48.4 (18.7)	66.6 (16.3)	66.6 (16.3)
Female: *n* (%)	4,672,589 (55.7)	19,947 (61.6)	89,217 (55.1)	2,885 (48.1)	16,358 (54.5)
Care-dependency level (1-5)	1,071,558 (12.8)	3,861 (11.9)	15,195 (9.4)	1,839 (30.7)	7,153 (23.9)
Administrative region 1: large cities: *n* (%)	1,830,585 (21.8)	6,845 (21.1)	34,225 (21.1)	1,405 (23.4)	7,025 (23.4)
Administrative region 2: urban district: *n* (%)	3,146,281 (37.5)	16,582 (51.2)	82,910 (51.2)	2,527 (42.1)	12,635 (42.1)
Administrative region 3: rural district with growing population density: *n* (%)	1,826,555 (21.8)	5,149 (15.9)	25,745 (15.9)	1,070 (17.8)	5,350 (17.8)
Administrative region 4: sparsely populated rural district: *n* (%)	1,589,129 (18.9)	3,802 (11.7)	19,010 (11.7)	996 (16.6)	4,980 (16.6)
Risk factors for severe COVID-19 course based on 35 groups of diseases, according to Roessler^b^: *n* (%)					
Obesity	624,974 (7.4)	1,559 (4.8)	8,813 (5.4)	644 (10.7)	3,647 (12.2)
Congenital immunodeficiency	16,688 (0.2)	78 (0.2)	394 (0.2)	27 (0.5)	104 (0.3)
Asthma	301,974 (3.6)	994 (3.1)	4,569 (2.8)	313 (5.2)	1,700 (5.7)
Autoimmune diseases	838,701 (10.0)	2,594 (8.0)	13,106 (8.1)	625 (10.4)	3,259 (10.9)
Hypertension	4,061,206 (48.4)	8,042 (24.8)	39,838 (24.6)	3,502 (58.4)	19,526 (65.1)
Ulcerative colitis	50,933 (0.6)	166 (0.5)	912 (0.6)	45 (0.8)	247 (0.8)
Dementia	366,828 (4.4)	1,861 (5.7)	8,449 (5.2)	711 (11.9)	3,410 (11.4)
Depression	795,923 (9.5)	2,853 (8.8)	13,511 (8.3)	854 (14.2)	3,809 (12.7)
Diabetes mellitus	1,221,888 (14.6)	2,185 (6.7)	10,420 (6.4)	1,407 (23.5)	7,214 (24.1)
Dialysis	8,804 (0.1)	196 (0.6)	728 (0.4)	179 (3.0)	895 (3.0)
Down syndrome	8,610 (0.1)	50 (0.2)	211 (0.1)	24 (0.4)	123 (0.4)
HIV/AIDS	13,315 (0.2)	51 (0.2)	220 (0.1)	11 (0.2)	55 (0.2)
Hepatitis	50,827 (0.6)	180 (0.6)	1,046 (0.6)	48 (0.8)	236 (0.8)
Heart failure	836,920 (10.0)	1,669 (5.2)	8,363 (5.2)	1,164 (19.4)	6,079 (20.3)
Immunocompromising diseases	250,903 (3.0)	742 (2.3)	4,258 (2.6)	375 (6.3)	1,892 (6.3)
Immunosuppressive therapy	207,340 (2.5)	548 (1.7)	2,876 (1.8)	258 (4.3)	1,230 (4.1)
Intellectual Disability	116,400 (1.4)	456 (1.4)	2,019 (1.2)	152 (2.5)	642 (2.1)
Interstitial lung disease	25,696 (0.3)	62 (0.2)	334 (0.2)	48 (0.8)	227 (0.8)
Coronary heart disease (CHD)	1,174,048 (14.0)	1,975 (6.1)	9,471 (5.9)	1,306 (21.8)	7,158 (23.9)
Metastasised solid tumours with therapy	26,555 (0.3)	40 (0.1)	248 (0.2)	49 (0.8)	218 (0.7)
Metastasised solid tumours w/o therapy	46,372 (0.6)	94 (0.3)	510 (0.3)	54 (0.9)	295 (1.0)
Morbus Crohn	42,660 (0.5)	137 (0.4)	799 (0.5)	26 (0.4)	137 (0.5)
Neurological diseases	694,622 (8.3)	2,163 (6.7)	9,938 (6.1)	893 (14.9)	4,172 (13.9)
Rheumatic diseases	444,979 (5.3)	1,039 (3.2)	5,193 (3.2)	375 (6.3)	2,443 (8.1)
Severe mental/psychiatric disorders	118,655 (1.4)	432 (1.3)	1,873 (1.2)	140 (2.3)	585 (2.0)
Solid cancers with therapy	63,035 (0.8)	117 (0.4)	601 (0.4)	91 (1.5)	419 (1.4)
Solid cancers w/o therapy	656,473 (7.8)	1,346 (4.2)	6,165 (3.8)	631 (10.5)	3,330 (11.1)
Atrial fibrillation and flutter	676,964 (8.1)	1,283 (4.0)	6,036 (3.7)	882 (14.7)	4,706 (15.7)
Cerebrovascular diseases	875,065 (10.4)	1,971 (6.1)	9,083 (5.6)	1,030 (17.2)	5,399 (18.0)
Cirrhosis and severe hepatic diseases	72,979 (0.9)	150 (0.5)	775 (0.5)	77 (1.3)	422 (1.4)
Health status after transplant	13,474 (0.2)	52 (0.2)	291 (0.2)	34 (0.6)	165 (0.6)
Chronic renal insufficiency	936,263 (11.2)	1,948 (6.0)	9,130 (5.6)	1,402 (23.4)	7,217 (24.1)
Chronic obstructive pulmonary disease (COPD) and other severe pulmonary diseases	515,325 (6.1)	1,216 (3.8)	5,769 (3.6)	607 (10.1)	3,232 (10.8)
Haemato-oncological diseases with therapy	14,336 (0.2)	27 (0.1)	159 (0.1)	35 (0.6)	169 (0.6)
Haemato-oncological diseases w/o therapy	60,086 (0.7)	135 (0.4)	710 (0.4)	76 (1.3)	376 (1.3)

^a^ Insured persons aged 18-99 years if they had been insured with the AOK without interruption during the pre-observation period until the start of post-observation period and were then continuously insured either for the entire follow-up period or until their death (if this occurred before that); had visited their general practitioner in April/May 2020 and did not have any COVID-19 diagnosis.

^b^ Roessler M, Jacob J, Risch L, Tesch F, Enders D, Wende D, et al. Hierarchisierung von Risikofaktoren für schwere COVID-19-Erkrankungsverlaeufe im Kontext der COVID-19-Schutzimpfungen. 2021(19):3–12.

### 3.2. Descriptive results and logistic regression

**Figure 2:**
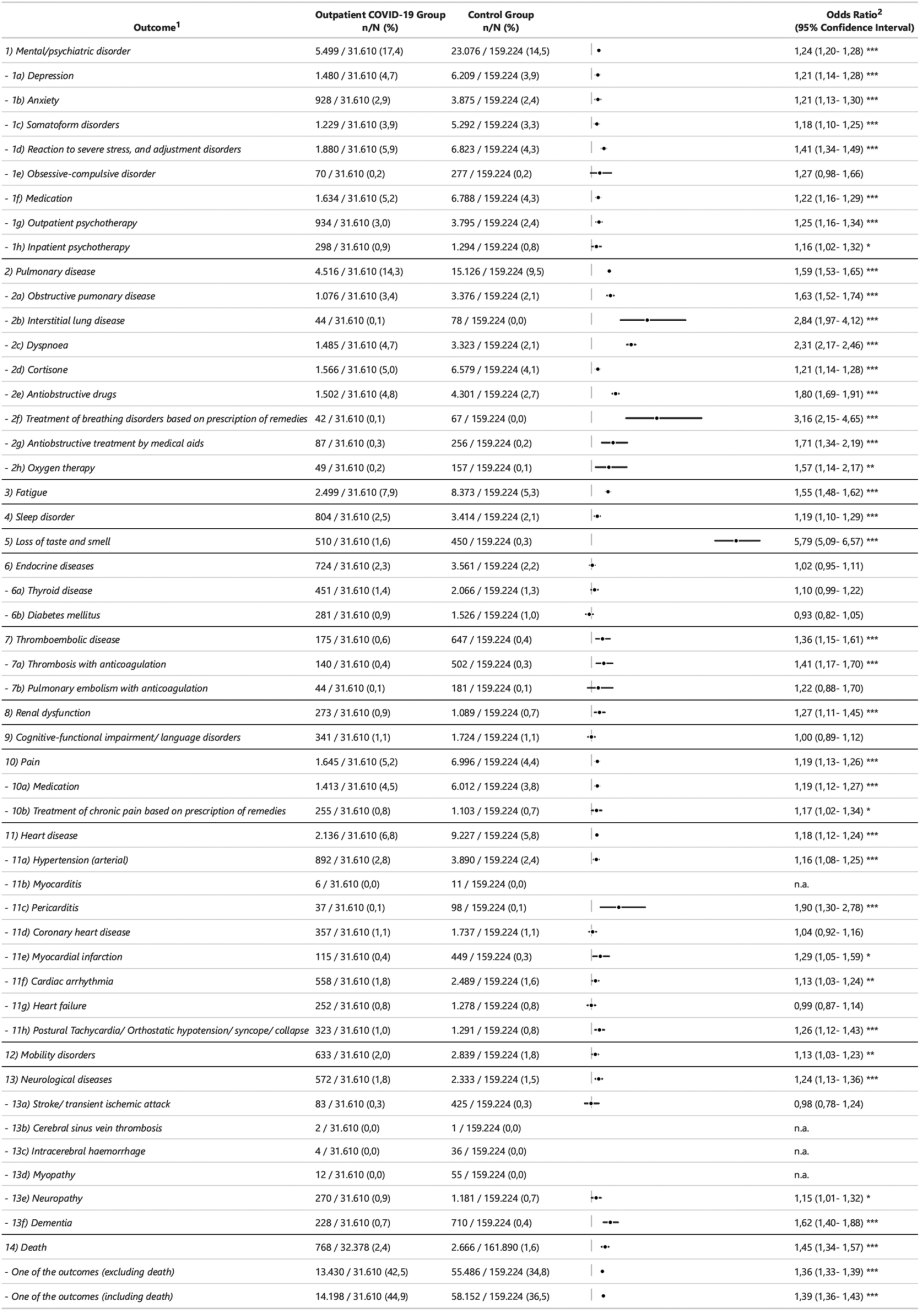
Descriptive results and odds ratios of outcome variables in the outpatient sample. ^1^ Operationalisation of the outcome variables is described in the supplement 1. ^2^ OR = Odds Ratio; CI = Confidence Interval; * *p* <= 0.05; ** *p* <= 0.01; *** *p* <= 0.001; n.a. = not assessed when value <10 (also within the sex categories).

**Figure 3:**
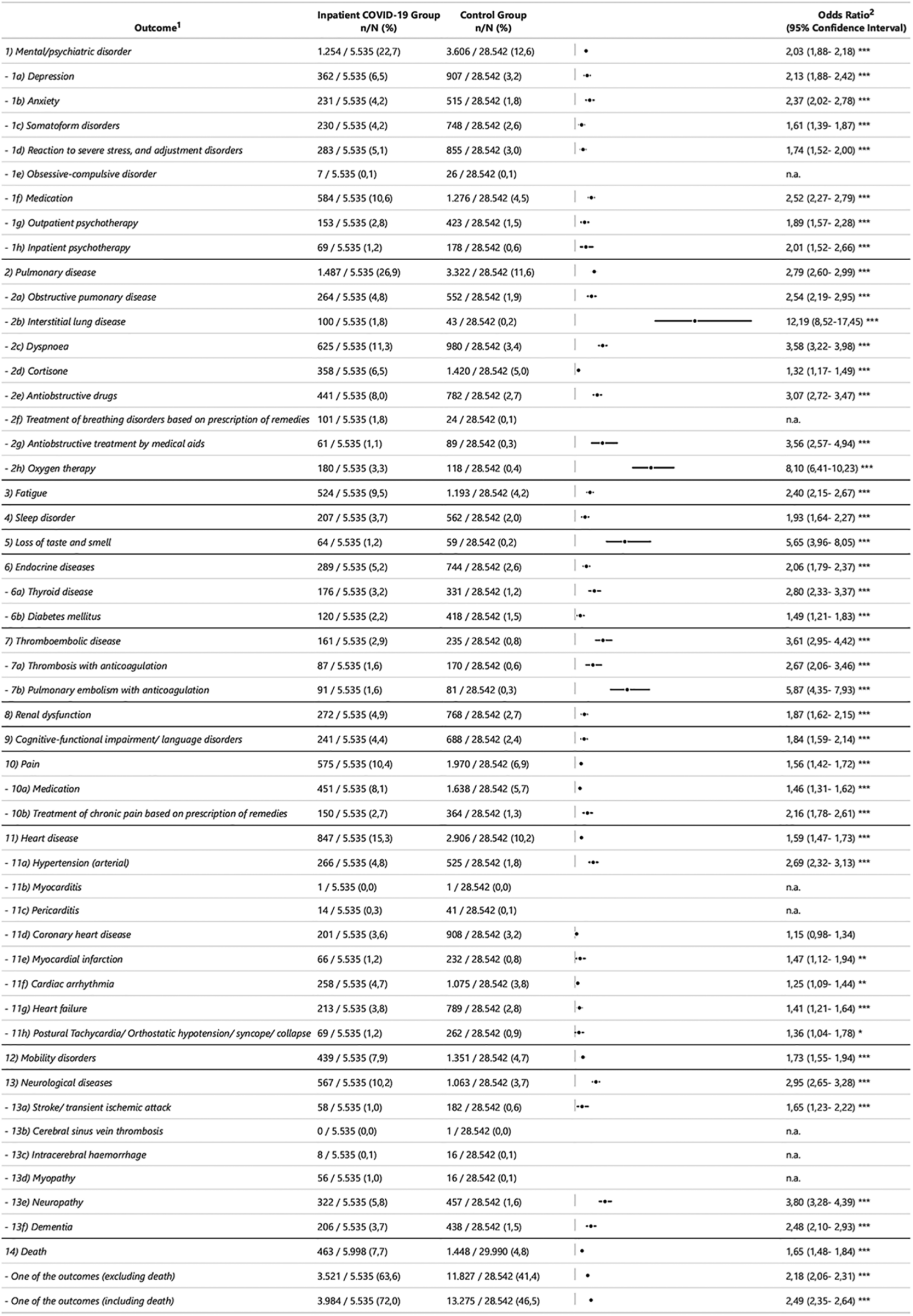
Descriptive results and odds ratios of outcome variables in the inpatient sample. ^1^ Operationalisation of the outcome variables is described in the supplement 1. ^2^ OR = Odds Ratio; CI = Confidence Interval; * *p* <= 0.05; ** *p* <= 0.01; *** *p* <= 0.001; n.a. = not assessed when value <10 (also within the sex categories).

**Figure 4:**
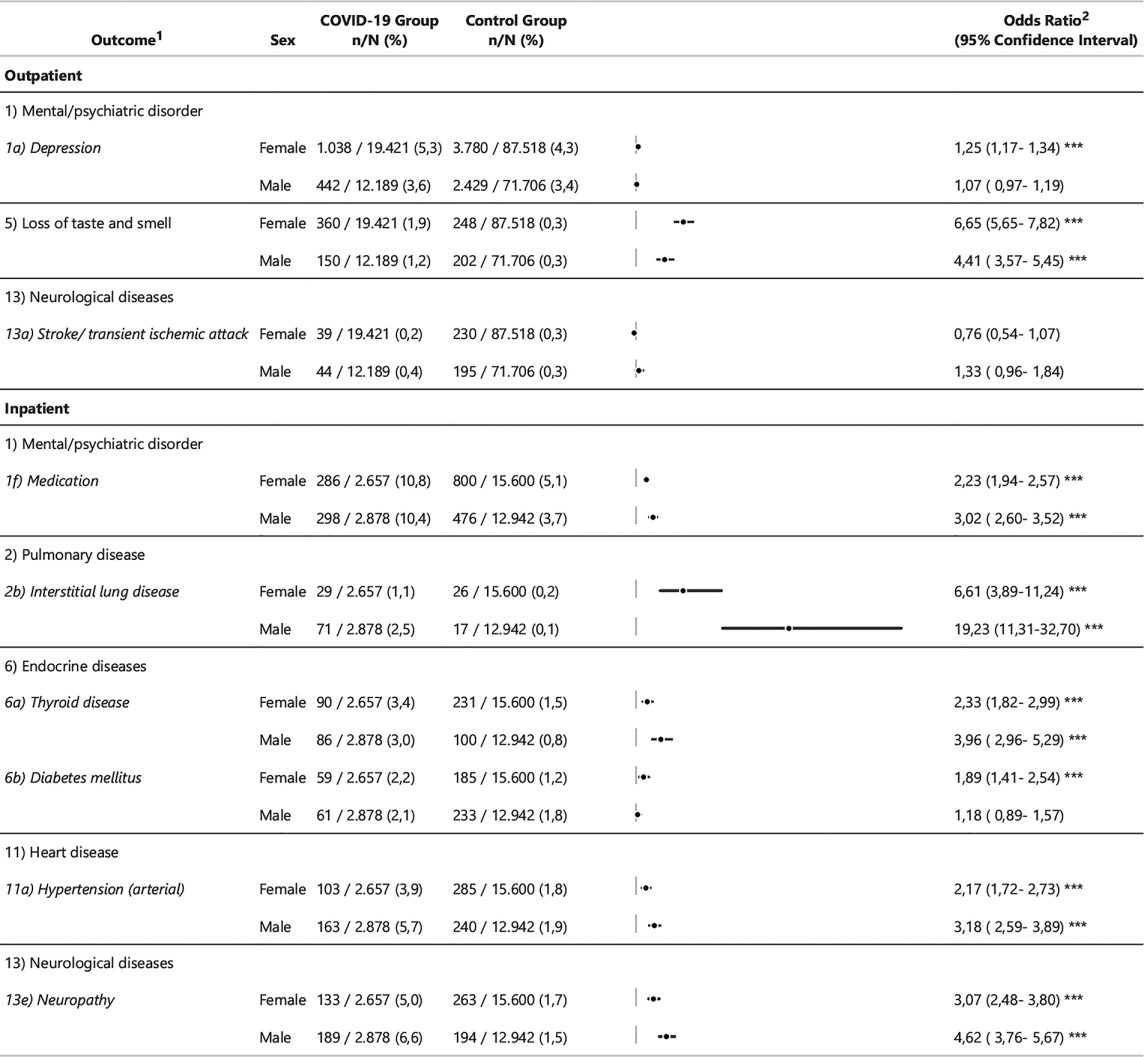
Descriptive results and odds ratios of outcome variables showing significant sex differences in the outpatient and the inpatient sample. ^1^ Operationalisation of the outcome variables is described in the supplement 1. ^2^ OR = Odds Ratio; CI = Confidence Interval; * *p* <= 0.05; ** *p* <= 0.01; *** *p* <= 0.001; n.a. = not assessed when value <10 (also within the sex categories).

Figures 2,3 show an overview of the descriptive results and regression models calculated independently of sex. Figure 4 shows the models that revealed a significant effect of the interaction of sex*COVID-19 in the second step of the logistic regression.

The odds ratio (OR) of developing post-acute treated health outcomes at the disease, symptom or treatment level in the 12 months following COVID-19 is increased for 42 of 50 (COVID-19 inpatient group) and 37 of 50 (COVID-19 outpatient group) outcomes compared to the control group. With regard to most outcome variables, people who were treated as inpatients for COVID-19 are more affected. That at least one of the outcome variables occurs in an individual is more frequently the case in both the outpatient and inpatient COVID-19 group compared to their control groups (OR = 1.4, 95%CI [1.4, 1.4]; OR = 2.5, 95%CI [2.4, 2.6]). Mortality is increased with OR of 1.7 [95%CI (1.5, 1.8), inpatient] and OR of 1.5 [95%CI (1.3, 1.6), outpatient].

Outpatients have the highest ORs in the main categories loss of smell and taste [OR = 5.8, 95%CI (5.1, 6.6)], pulmonary diseases [OR = 1.6, 95%CI (1.5, 1.7)] and fatigue [OR = 1.6, 95%CI (1.5–1.6)]. In the sub-categories interstitial respiratory diseases [OR = 2.8, 95%CI (2.0, 4.1)], dyspnoea [OR = 2.3, 95%CI (2.2, 2.5)] and treatment of breathing disorders by prescribing remedies [OR = 3.2, 95%CI (2.2, 4.7)] have the highest ORs. As shown in Figure 2, the low rate of occurrence for some outcome variables must be taken into consideration, e.g., for interstitial pulmonary diseases (0.1% (outpatient COVID-19 group); 0.0% (control group)), loss of smell and taste (1.6%; 0.3%) or thromboses (0.4%; 0.3%). No evident differences are seen in the outpatient group in the main categories endocrinal diseases (incl. sub-categories), cognitive functional impairments/language disorders as well as in few sub-categories: obsessive-compulsive disorders, pulmonary embolism with anticoagulation, coronary heart disease, heart failure, transient ischaemic attacks and stroke. Because of the low number of cases, myocarditis, cerebral sinus vein thrombosis, intracerebral haemorrhage and myopathy were not evaluated (Figure 2).

In the inpatient group, the highest ORs are shown in the main category pulmonary diseases in interstitial lung diseases [OR = 12.2, 95%CI (8.5, 17.5)], oxygen therapy [OR = 8.1, 95%CI (6.4, 10.2)], anti-obstructive therapy (medical aids) [OR = 3.6, 95%CI (2.6, 4.9)], dyspnoea [OR = 3.6, 95%CI (3.2, 4.0)] and anti-obstructive drugs [OR = 3.1, 95%CI (2.7–3.5)], as well as in in the main category thromboembolic diseases the pulmonary embolism with anticoagulation [OR = 5.9, 95%CI (4.4, 7.9)]. In the sub-category coronary heart disease there were no detectable differences in inpatients. Due to the small number of cases, it was not possible to analyse the subcategories obsessive-compulsive disorders, treatment of breathing disorders by prescribing remedies, myocarditis/pericarditis, cerebral sinus vein thrombosis, intracerebral haemorrhage, myopathy and neuropathy. As shown in Figure 3, the small number of cases for some outcomes is to be considered, for example, interstitial respiratory diseases (1.8% (inpatient COVID-19 group); 0.2% (control group)), loss of smell and taste (1.2%; 0.2%), thrombosis (1.6%; 0.6%) and pulmonary embolism (1.6%; 0.3%).

An increased OR of 1.6 [95%CI (1.4, 1.9)] was seen in the outpatient group for the diagnosis of a newly occurring dementia, however not for cognitive functional impairments and language disorders, while in the inpatient group, dementia [OR = 2.5, 95%CI (2.1, 2.9)] and cognitive functional impairments and language disorders [OR = 1.8, 95%CI (1.6, 2.1)] show an increased OR compared with the control group.

For individuals with outpatient management of acute COVID-19, significant differences between men and women are particularly rare: An increased OR could be seen among the women in the outpatient group for depression (women: OR = 1.3, 95%CI [1.2, 1.3]; men: OR = 1.1, 95%CI [1.0, 1.2]) as well as loss of smell and taste (women: OR = 6.7, 95%CI [5.7, 7.8]; men: OR = 4.4, 95%CI [3.6, 5.5]), while stroke/ transient ischaemic attacks show a higher OR in men in the outpatient group (men: OR = 1.3, 95%CI [1.0, 1.8]; women: OR = 0.8, 95%CI [0.5, 1.1]).

Among male compared to female individuals an increased OR is evident for the inpatient group in the categories psychiatric medication, interstitial respiratory diseases, thyroid diseases, hypertension and neuropathy (Figure 4). Only in the category diabetes mellitus is an increased OR shown for women compared to men.

To evaluate the possibility of distorted results, nursing dependency level was included in a sensitivity analysis as a proxy variable for a potentially increased pre-existing higher morbidity in the COVID-19 groups compared to the matched CGs (39). The odds ratios for outcomes relating to cardiac arrythmias, neuropathy, chronic pain, and mobility problems were attenuated slightly and the confidence intervals included 1. However, the 95% confidence interval for these outcome variables had already been very close to the value 1 beforehand. Furthermore, while the OR of the outcome variable death in both samples is still significantly increased, nevertheless it drops by circa 0.2 points (outpatient: OR = 1.2, 95%CI [1.1, 1.3]; inpatient: OR = 1.4, 95%CI [1.3, 1.6]). Therefore, conclusions relating to these variables should be interpreted cautiously.

## 4. Discussion

After a SARS-CoV-2 infection, there is an increased occurrence of post-acute health sequelae in both the outpatient and inpatient COVID-19 groups compared to the contemporaneous control groups, showing greater severity after inpatient courses. Similar results are shown in US data, but with stratification by age and without consideration of treatment by medication, therapies, or psychotherapy (30). At the symptom level, the prominent symptom complex described in the literature is confirmed in the areas of lung, neurocognition/mental health and fatigue (14, 22, 23). Mortality was increased compared to the control group (inpatient: OR = 1.7, 95%CI [1.5, 1.8], outpatient: OR = 1.5, 95%CI [1.3, 1.6]).

There is an increased probability of occurrence of diseases with potentially acutely serious course such as myocardial infarction, stroke/ transient ischaemic attack, thrombosis and pulmonary embolism (inpatient), as well as myocardial infarction and thrombosis (outpatient). This is compatible with findings on hypercoagulability in the acute COVID phase (31) and health sequelae of thrombo-embolic and cardio-ischaemic nature after COVID-19 (15, 16, 20, 32) and clearly shows a risk beyond the acute COVID-19 phase of 4 weeks. Even though these complications in absolute numbers are described only rarely, bearing in mind the frequency of persistent symptoms (dyspnoea, chest pain) and at the same time increased mortality (7.7% vs. 4.8% inpatient; 2.4% vs. 1.6% outpatient), the field of tension between underdiagnostic and overdiagnostic in the everyday clinical setting becomes visible.

Especially after inpatient COVID-19 courses, chronic diseases in need of treatment are seen to a greater extent. Continuous connectivity with a view to the whole person as well as risk-adapted controls (e.g., blood pressure, kidney values, indications of heart failure) appear to be a sensible medical measure here. The results show similarities to Ayoubkhani et al. (16).

Both outpatients and inpatients showed an increased OR for mental health disorders after COVID-19 compared to the control group. This is in line with other results from the literature (24, 33, 34); at the same time, there are also results for no (30) or a temporarily increased risk of affective disorders for patients under 65 years of age (34). The sex difference in post-acute health sequelae after COVID-19 is less pronounced than previous findings suggested (14, 35). After an inpatient COVID-19 acute course, men dominate in the sex-specific significantly different categories, which could be due to more severe acute courses in men (36).

### 4.1. Strengths and weaknesses

The evaluation of all routine data of the AOKs results in a comprehensive database that includes more than 30% of the German resident population and comprises of utilization information regarding the majority of health care services in Germany. Limitations arise because routine data of the statutory health insurance do not show morbidity as such, but (I) treated and (II) documented morbidity after (III) insured persons have sought medical help, (IV) have explicitly mentioned their disease and (V) it has been correctly coded. Other possible biases could be: under- or over-reporting at the symptom level; residual confounding, lack of specific coding options (e.g., post exertional malaise, cognitive impairment); too narrow a grid of the selected outcome diseases (e.g., autoimmune diseases, rheumatic diseases as well as vasculitides are hardly recorded); under-reporting/distortion of the pandemic situation in the index period (possible non-testing bias in the control group due to changed utilisation behaviour in the first wave, leading to the possibility of individuals with an undiagnosed/undocumented SARS-CoV-2 infection being assigned to the control group); overestimation of the morbidity of the inpatient COVID-19 group due to hospitalisation. The underlying sample of AOK-insured persons corresponds to about one third of the total population and is by that representative for the German population in age and gender. However, a limitation of the representativeness of the population of the CG could have been introduced by the matching process, as controls were individuals who 1. visited their general practitioner in April/May 2020, 2. had no hospitalisation in April to June 2020 and 3. had no COVID-19-related diagnosis during the entire observation period. As the results are derived from data from the first wave of the pandemic, it can be assumed that both vaccination and changes in virus variants would alter analyses in subsequent periods. Current evidence suggests a lower probability of post-COVID after vaccination and after illness with the Omicron subtypes (37, 38).

### 4.2. Conclusions

In the routine data of the statutory health insurance, a very broad spectrum of post-acute treated health sequelae can be seen under the umbrella term “long-/post-COVID”, including cardiac, neurological, psychological and thromboembolic diseases, fatigue, lung diseases and kidney function disorders. The variety of symptoms and diagnoses requires an interdisciplinary and interprofessional cooperation among general practitioners, medical specialists, psychotherapists and healthcare providers offering medical remedies.

Following a SARS-CoV-2 infection during the first wave of the COVID-19 pandemic in Germany, 8.4 [COV-OUT, 95%CI (7.7, 9.1)] respectively 25.5 [COV-IN, 95%CI (23.6, 27.4)] percentage points more subjects showed at least one new diagnosis/symptom/treatment compared to their matched CG (COV-OUT: 44.9%, CG: 36.5%; COV-IN: 72.0%, CG: 46.5%). The results reinforce evidence of an increased burden on the health care system from increased utilization due to post-acute sequelae after COVID-19 (40, 41). Further surveys should evaluate efficient, coordinated care pathways with consideration of patient-related outcomes such as quality of life, social participation, health care system resources, and economics (42).

## Data Availability

The data analyzed in this study is subject to the following licenses/restrictions: Unfortunately, the data cannot be released. Since these data are not aggregated, but are available at the insured person level and are included in the analyses at this level, it is not possible to pass them on in terms of data protection. Requests to access these datasets should be directed to Doreen.Mueller@wido.bv.aok.de.
