# Supplementary material for: Treated post-acute sequelae after COVID-19 in a German matched cohort study using routine data from 230,256 adults
Source: Front Epidemiol. 2023 Feb 14;2:1089076. doi: 10.3389/fepid.2022.1089076 (PMC10911009; doi:10.3389/fepid.2022.1089076)
Supplement: Supplementary file 1 [file Table1.docx]

Supplementary Material

**Supplementary Table 1: Operationalisation of outcome parameters**

| **Outcome parameters** | **Operationalisation ^1,2^** |
| --- | --- |
| 1) Mental/psychiatric disorder | At least one of the parameters 1a-h is fulfilled |
| 1a) Depression | At least one of the diagnoses F32, F33, F34.1 (outpatient (general practitioner or specialist groups trained in psychodiagnosis) or inpatient or ambulatory psychiatric services in hospitals) |
| 1b) Anxiety | One of the diagnoses F40 F41 F06.4 (outpatient (general practitioner or specialist groups trained on psychodiagnosis) or inpatient or ambulatory psychiatric services in hospitals) |
| 1c) Somatoform disorders | Diagnosis F45 (outpatient (general practitioner or specialist groups trained on psychodiagnosis) or inpatient or ambulatory psychiatric services in hospitals) |
| 1d) Reaction to severe stress, and adjustment disorders | Diagnosis F43 (outpatient (general practitioner or specialist groups trained on psychodiagnosis) or inpatient or ambulatory psychiatric services in hospitals) |
| 1e) Obsessive-compulsive disorder | Diagnosis F42 (outpatient (general practitioner or specialist groups trained on psychodiagnosis) or inpatient or ambulatory psychiatric services in hospitals) |
| 1f) Medication | At least one of the drug prescriptions N05A, N05B, N06A |
| 1g) Outpatient psychotherapy | Outpatient care (all services described on PFS section 35 (Psychotherapy guideline) subject to application, inclusive probatory sessions, consultation, acute care) or treatment in ambulatory psychiatric services in hospitals |
| 1h) Inpatient psychotherapy | Inpatient care with a discharge diagnosis included in ICD chapter V (Mental and behavioural disorder) |
| 2) Pulmonary disease | At least one of the parameters 2a-h is fulfilled |
| 2a) Obstructive pumonary disease | At least one of the diagnoses J43, J44, J45, J46, J47 (outpatient or inpatient) |
| 2b) Interstitial lung disease | Diagnosis J84 (outpatient or inpatient) |
| 2c) Dyspnoea | Diagnosis R06.0 (outpatient or inpatient) |
| 2d) Cortisone | Drug prescription H02AB |
| 2e) Antiobstructive drugs | At least one of the drug prescriptions R03A, R03B |
| 2f) Treatment of breathing disorders based on prescription of remedies | At least one therapy prescription for physiotherapy treatments – diagnosis group AT1, AT2 according to the therapy guideline |
| 2g) Antiobstructive treatment by medical aids | Medical aid prescription for an aerosol inhalation device for the lower respiratory tract |
| 2h) Oxygen therapy | At least one the device prescriptions for oxygen therapy equipment: filling unit for compressed gas, oxygen concentrator, compressed and liquid gas |
| 3) Fatigue | At least one of the diagnoses G93.3, F48.0, R53 (outpatient or inpatient) |
| 4) Sleep disorder | At least one of the diagnoses G47.01, G47.02, G47.9, F51 (outpatient) |
| 5) Loss of taste and smell | Diagnosis R43 (outpatient or inpatient) |
| 6) Endocrine diseases | At least one of the parameters 6a-b is fulfilled |
| 6a) Thyroid disease | At least one of the drug prescriptions H03B, H03A |
| 6b) Diabetes mellitus | At least one of the diagnoses E11, E12, E13, E14 (outpatient) and at least one of the drug prescriptions A10A, A10B |
| 7) Thromboembolic disease | At least one of the parameters 7a-b is fulfilled |
| 7a) Thrombosis with anticoagulation | At least one of the diagnoses I80.1, I08.2, I80.3, I80.8, I80.9, I81, I82 (outpatient or inpatient) and at least one of the drug prescriptions B01AA, B01AB, B01AE, B01AF |
| 7b) Pulmonary embolism with anticoagulation | Diagnosis I26 (outpatient or inpatient) and at least one of the drug prescriptions B01AA, B01AB, B01AE, B01AF |
| 8) Renal dysfunction | At least one of the diagnoses N17, N18.1-5, N18.8, N18.9, N19 or worsening when N18 (outpatient or inpatient) |
| 9) Cognitive-functional impairment/ language disorders | At least one of the diagnoses F06.7, U51, R47 (outpatient or inpatient) |
| 10) Pain | At least one of the parameters 10a-b is fulfilled |
| 10a) Medication | At least one of the drug prescriptions N02A, N03AX16, N03AX12 |
| 10b) Treatment of chronic pain based on prescription of remedies | At least one of the therapy prescriptions physiotherapy treatments - diagnosis group chronic pain syndrome according to the therapy guideline |
| 11) Heart disease | At least one of the parameters 11a-h is fulfilled |
| 11a) Hypertension (arterial) | At least one of the diagnoses I10, I11, I12, I13 (outpatient) and at least one of the drug prescriptions C03, C07, C08, C09  OR at least one of the diagnoses I10, I11, I12, I13 (outpatient) pre-existing and at least one of the drug prescriptions C03, C07, C08, C09 new |
| 11b) Myocarditis | At least one of the diagnoses I40, I41 (outpatient-cardiology or inpatient) |
| 11c) Pericarditis | At least one of the diagnoses I30 I31.0, I31.1, I31.2, I31.3, I32 (outpatient-cardiology or inpatient) |
| 11d) Coronary heart disease | At least one of the diagnoses I20.1, I20.8, I24.0, I24.8, I24.9, I25 (outpatient-cardiology or inpatient) |
| 11e) Myocardial infarction | At least one of the diagnoses I21, I22, I23, I24.1 (outpatient) or diagnosis I20.0 (outpatient or inpatient surgery) |
| 11f) Cardiac arrhythmia | At least one of the diagnoses I47, I48, I49 (outpatient-cardiology or inpatient) |
| 11g) Heart failure | At least one of the diagnoses I50.03, I50.04, I50.05, I50.12, I50.13, I50.14, I13 (outpatient-cardiology or inpatient) |
| 11h) Postural Tachycardia/ Orthostatic hypotension/ syncope/ collapse | At least one of the diagnoses I95.1, R55 (outpatient) |
| 12) Mobility disorders | At least one of the diagnoses R26.2, R26.3, R29.6, Z99.3, Z74.0 (outpatient or inpatient) |
| 13) Neurological diseases | At least one of the parameters 13a-f is fulfilled |
| 13a) Stroke/ transient ischemic attack | At least one of the diagnoses I63, I64, I66, G45, G46 (inpatient) |
| 13b) Cerebral sinus vein thrombosis | Diagnosis I67.6 (inpatient) |
| 13c) Intracerebral haemorrhage | At least one of the diagnoses I61, I62 (inpatient) |
| 13d) Myopathy | At least one of the diagnoses G72.4, G72.8, G72.9, G73.4 (outpatient or inpatient) |
| 13e) Neuropathy | At least one of the diagnoses G62.8, G62.9, G63.0 (outpatient or inpatient) |
| 13f) Dementia | At least one of the diagnoses F00, F01, F02, F03, G30 (outpatient or inpatient) |
| 14) Death | Died during post-observation period |

**^1^** Diagnosis codes according to ICD-10-GM; drug prescriptions according to ATC code; diagnosis groups in accordance with the therapy guideline (BAnz AT 07.04.2022 B2); codes specified without end position refer to all possible end position codes in each case

^2^ Incident diagnoses were recorded. That indicates whether an event occurred for the first time in the post-observation period compared with the pre-observation period or whether it worsened, in the case of kidney failure or hypertension
